# Splice implantation of multiple drug-eluting stents for tandem severe stenosis of the internal carotid artery: A case report

**DOI:** 10.1097/MD.0000000000037561

**Published:** 2024-03-22

**Authors:** Qinghai Dai, Lingfeng Shu, Pengcheng Zhu, Hongtu Tan, Tao Wu

**Affiliations:** a Department of Interventional Medicine, Brain Disease Hospital, The First Affiliated Hospital of Henan University of Chinese Medicine, Zhengzhou, China; b The First Clinical Medical College, Henan University of Chinese Medicine, Zhengzhou, China.

**Keywords:** drug-eluting stent, endovascular therapy, internal carotid artery tandem stenosis, ischemic stroke

## Abstract

**Rationale:**

Severe stenosis of the internal carotid artery tandem affects the blood supply to the brain and threatens human life, which can be solved by interventional procedures.

**Patient concerns:**

A 64-year-old male patient presented with a sudden onset of dizziness, palpitation, numbness, and weakness of the limbs. Imaging studies suggested multiple tandem severe stenoses from the left internal carotid artery contrast C2 to C4.

**Diagnosis:**

Severe stenosis of the left internal carotid tandem.

**Interventions:**

Multiple drug-eluting stent splicing and implantation were performed.

**Outcomes:**

The left internal carotid artery stenosis was released, intracranial vascular filling was significantly improved, and the patient recovered well.

**Lessons:**

Interventional implantation of multiple drug-eluting stents relieves tandem severe stenosis of the internal carotid artery, with a wide range of applicability, high safety profile, and rapid postoperative recovery compared with endothelial debridement procedures.

## 1. Introduction

Cerebrovascular stenosis is the main cause of ischemic stroke, which is mostly caused by atherosclerosis in the elderly.^[1]^ While in young people, factors causing cerebrovascular stenosis mainly include vascular dysplasia, vasculitis, coagulation dysfunction, and genetic diseases.^[2]^ Cerebrovascular tandem severe stenosis has the most serious impact on the human body, increasing the risk of cerebral tissue hypoperfusion, with poor pharmacological treatment and difficult surgical procedures.^[3]^

## 2. Case presentation

**Figure 1.**
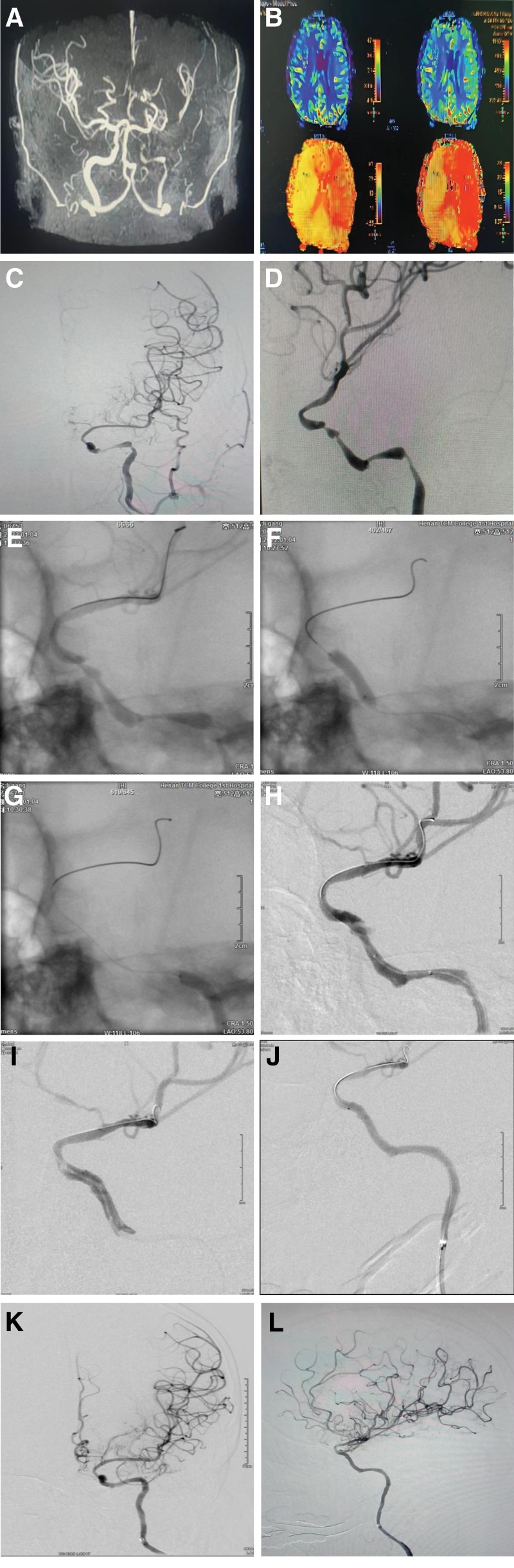
(A) MRA showed severe stenosis of the left internal carotid tandem; occlusion of the left anterior cerebral artery and severe stenosis of the left middle cerebral artery. (B) CTP suggested that the left side of the brain had ischemia-reperfusion alterations. (C, D) Left internal carotid artery with severe stenosis from C2 to C4 and poor visualization of the left anterior cerebral artery on orthopantomography and lateral angiography. (E) Intraoperative microguide placed in M3 segment. (F–H) Low-pressure balloon dilatation from C4 to C2 segments, stenosis lifted, clamping in C4 segments. (I) Stent implanted in the C4 segment with perfect apposition to the vessel wall and disappearance of the entrapment. (J) Third stent implanted in C2 segment with well-placed splice. (K, L) Postoperative internal carotid artery orthopantomography and lateral angiography with release of stenosis and significant improvement in intracranial vascular filling compared to the preoperative period.

**Figure 2.**
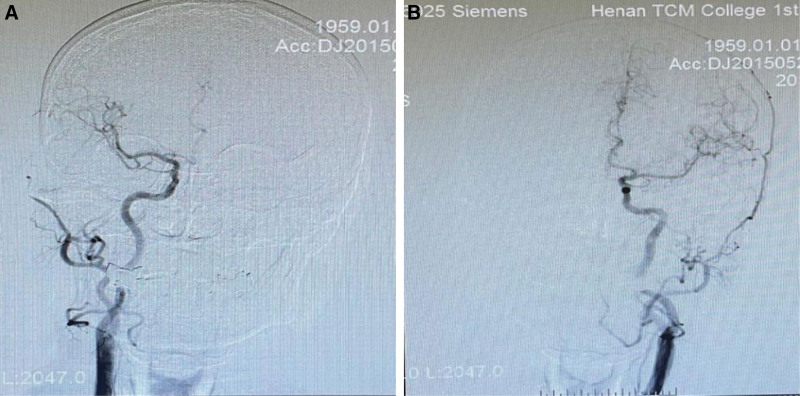
(A and B) Past history DSA angiography showed good cerebral vascular filling and no abnormalities.

The male patient, 64 years old, presented with “sudden onset of dizziness, palpitation, and numbness and weakness of the limbs for 2 hours.” The previous history of hypertension, hypertension has the highest 200/100 mm Hg, nifedipine 20 mg po qd, daily blood pressure control is about 135/86 mm Hg. The patient was admitted to the hospital on December 17, 2021. Physical examination: clear consciousness, unfavorable speech, a slightly rightward deviation of the angle of the mouth, a lightening of the frontal line on the left side, muscle strength of Grade 4 in the right upper limb, muscle strength of other limbs was normal, muscle tension was unchanged. NIHSS: 2 scores. Cranial MRA showed intracranial atherosclerotic changes; tandem severe stenosis of the left internal carotid artery; occlusion of the left anterior cerebral artery and severe stenosis of the left middle cerebral artery (Fig. 1A). CTP suggested that the left lateral paraventricular mean blood flow CBF, mean blood volume CBV were elevated compared with the contralateral side, peak time MTT, mean transit time TTP were increased compared with the right side, and the left side of the brain had ischemia-reperfusion alterations (Fig. 1B). DSA showed multiple tandem severe stenoses from the left carotid artery contrasting C2(petrous segment) to C4(cavernous segment), with a degree of stenosis of about 90%; the left anterior cerebral artery did not appear to be visible; (Fig. 1C and D). Laboratory tests were normal. In 2015, the patient was admitted to our hospital with “dizziness and weakness in the limbs for a week.” DSA examination showed good vessel visualization without stenosis (Fig. 2A and B). Considering the rapid progression of the patient’s disease and the severe stenosis of tandem vessels, multiple drug-eluting stent implantation in the ICA was proposed to be performed on January 10, 2022,

The patient took the supine position, and under general anesthesia, the 6F long sheath was over-selected to the distal common carotid artery under the cooperation of 5FMAP1 catheter, and the 5F distal access catheter was placed in the cervical segment of the left internal carotid artery, and the tandem severe stenosis of the left internal carotid artery from the C2 to C4 was observed by contrast and the stenosis was more than 90%. The microguide wire (ASAHI0, 014 * 200 cm) and 1.7F microcatheter were inserted to pass through the tandem stenosis and then placed in the M3 of the middle cerebral artery (Fig. 1E), with the head end of the implanted exchange microguide wire (ASAHI0, 014 * 300 cm) molded in the form of a pig’s tail. A low-pressure balloon (NeuroLPS, 3.0 * 15 mm) was implanted and predilated from the distal end to the proximal end of the stenosis of the internal carotid artery. Radiography showed that there was dissection in the C4 segment and the stenosis was relieved (Fig. 1F–H). The balloon catheter was anchored in the ophthalmic segment of the ICA, and the access catheter followed to the C4 segment near the lesion, and the first drug-eluting stent (NOVA, 3.0 * 15 mm) was placed in the distal stenosis to be released at a named pressure, and the contrast showed that the drug-eluting stent was well opposed to the wall, and the entrapment disappeared (Fig. 1I). The drug-eluting stent (NOVA, 3.0 * 20 mm) was placed again in the C3 segment, and the head end of the drug-eluting stent was overlapped with the tail end of the previous drug-eluting stent for 3 mm, and the drug-eluting stent was released at a named pressure. The intermediate catheter was withdrawn and the drug-eluting stent (NOVA, 3.5 * 15 mm) was placed in the C2, the head end of the drug-eluting stent coincided with the tail end of the second drug-eluting stent, and the drug-eluting stent was released at 6 standard atmospheric pressure. The balloon catheter was retracted to the middle of the third drug-eluting stent, and the caudal end of stent was released into a cone-like shape at 14 standard atmospheric pressure, and the caudal end of the drug-eluting stent was completely attached to the wall (Fig. 1J). Postoperative imaging showed that the left internal carotid artery stenosis was relieved, the left anterior cerebral artery was well visualized, and the intracranial vascular filling was significantly improved compared with before (Fig. 1K and L). The patient’s neurological examination after awakening from anesthesia was not abnormal.

Telephone follow-up was performed for 3 months, 6 months, 1 year, and to date. The patient did not experience any cerebral ischemic events and had normal limb movement and speech function.

## 3. Discussion

The patient had favorable cerebral vascular filling with no significant stenosis on imaging 6 years ago, but severe series stenosis of the left internal carotid artery was found in this examination. Indicating that the stenosis progressed rapidly and there were lesions in the vessel wall, and there was a high risk of restenosis after implantation of bare metal drug-eluting stents. Self-expanding stents may have a long stenosis span and insufficient pressure release, resulting in poor apposition of the stent to the vessel wall and increasing the risk of postoperative stent migration.^[4,5]^ One self-expanding stent does not completely cover the stenotic segment, and several stents still need to be implanted in a stitched fashion. Therefore, drug-eluting stents were chosen to be implanted into the stenotic segment of the left internal carotid artery for the long-term benefit of the patient.

Patients with internal carotid artery stenosis are mostly treated with both carotid endarterectomy and interventional treatment. In this case, the tandem severe stenosis of the internal carotid artery from the C2 to the C4 segments in our patient, surgical exposure of the diseased vessel was difficult, and the endothelium could not be stripped off. Therefore, interventional therapy was the most suitable choice.^[6]^ During the operation, a low-pressure balloon was used to predilate the severe stenosis segment of the internal carotid artery from the distal to proximal to facilitate drug-eluting stent implantation. Vascular dissection appears in C4 segment after balloon dilatation, which may lead to intimal tear due to ulceration of the vessel wall and plaque rupture.^[7]^ It also proves that there are lesions in the intima of the patients, which leads to the rapid progress of ICA stenosis. When drug-eluting stents are implanted, we choose to give priority to the treatment of distal carotid artery stenosis. To prevent priority treatment of the proximal end of the internal carotid artery, the inability of distal drug-eluting stents to pass smoothly, and the occurrence of proximal stent slippage.

The choice of drug-eluting stent type is based on the anatomical shape of the operated vessel and the need for overlapping splices. The second drug-eluting stent was chosen to be longer than the other 2 drug-eluting stents to provide complete coverage of the mid-stenosis and to interconnect with the other 2 drug-eluting stents. Because the proximal end of the internal carotid artery is thicker than the distal end, the diameter of the third drug-eluting stent is larger than that of the first 2. According to the instructions for use, an increase in standard named pressure of 6 atmospheres will expand the standard diameter of the stent. Thus, expanding the inner diameter of the second stent so that the head end of the third drug-eluting stent meets the end of the second drug-eluting stent. Moreover, releasing the tail end of the third drug-eluting stent with 14 standard atmospheric pressure made the tail end of the drug-eluting stent cone-like, which ensured that the tail end of the drug-eluting stent fits closely to the proximal end of the ICA.

## 4. Conclusion

implantation of multiple drug-eluting stents can bring good therapeutic effects for patients with severe series stenosis of the internal carotid artery.

## Author contributions

**Writing—original draft:** Qinghai Dai.

**Formal analysis:** Lingfeng Shu.

**Visualization:** Pengcheng Zhu.

**Project administration:** Hongtu Tan.

**Writing—review & editing:** Tao Wu.
